# Assessing readiness to implement long-acting injectable HIV antiretroviral therapy: provider and staff perspectives

**DOI:** 10.1186/s43058-023-00506-3

**Published:** 2023-10-19

**Authors:** Kimberly A. Koester, Jonathan A. Colasanti, Moira C. McNulty, Kaylin Dance, Xavier A. Erguera, Manami Diaz Tsuzuki, Mallory O. Johnson, John A. Sauceda, Elizabeth Montgomery, John Schneider, Katerina A. Christopoulos

**Affiliations:** 1 Division of Prevention Science, University of California, San Francisco, 550 16th Street, San Francisco, CA 94105 USA; 2 Division of Infectious Diseases, Department of Medicine, Emory University School of Medicine, Atlanta, GA USA; 3 Chicago Center for HIV Elimination, University of Chicago, Chicago, USA; 4 Section of Infectious Diseases and Global Health, Department of Medicine, University of Chicago, Chicago, IL USA; 5 Division of HIV, ID and Global Medicine, Department of Medicine, University of California San Francisco, San Francisco, CA USA; 6 Women’s Global Health Imperative, RTI International, Berkeley, CA USA; 7 Department of Epidemiology and Biostatistics, School of Medicine, University of California San Francisco, San Francisco, CA USA

**Keywords:** Long-acting injectable medication, HIV antiretroviral therapy, Focus groups, Pre-implementation, CFIR, Qualitative methods

## Abstract

**Background:**

Long-acting injectable antiretroviral therapy (LAI-ART) represents the next innovation in HIV therapy. Pre-implementation research is needed to develop effective strategies to ensure equitable access to LAI-ART to individuals living with HIV.

**Methods:**

We conducted focus group discussions (FGDs) with providers and staff affiliated with HIV clinics in San Francisco, Chicago, and Atlanta to understand barriers to and facilitators of LAI-ART implementation. Participants also completed a short survey about implementation intentions. FGDs were held via video conference, recorded, transcribed, and thematically analyzed using domains associated with the Consolidated Framework for Implementation Research (CFIR).

**Results:**

Between September 2020 and April 2021, we led 10 FDGs with 49 participants, of whom ~60% were prescribing providers. Organizational readiness for implementing change was high, with 85% agreeing to being committed to figuring out how to implement LAI-ART. While responses were influenced by the unique inner and outer resources available in each setting, several common themes, including implementation mechanisms, dominated: (1) optimism and enthusiasm about LAI-ART was contingent on ensuring equitable access to LAI-ART; (2) LAI-ART shifts the primary responsibility of ART adherence from the patient to the clinic; and (3) existing clinic systems require strengthening to meet the needs of patients with adherence challenges. Current systems in all sites could support the use of LAI-ART in a limited number of stable patients. Scale-up and equitable use would be challenging or impossible without additional personnel. Participants outlined programmatic elements necessary to realize equitable access including centralized tracking of patients, capacity for in-depth, hands-on outreach, and mobile delivery of LAI-ART. Sites further specified unknown logistical impacts on implementation related to billing/payer source as well as shipping and drug storage.

**Conclusions:**

Among these HIV care sites, clinic readiness to offer LAI-ART to a subset of patients is high. The main challenges to implementation include concerns about unequal access and a recognition that strengthening the clinic system is critical.

Contributions to the literature

- Long-acting injectable antiretroviral therapy is an innovation in HIV treatment that requires patients to attend injection appointments every 4 or 8 weeks however clinics are not designed to accommodate a high volume of injection visits.
- Through focus group discussions, we assessed provider and staff perspectives on the acceptability, feasibility, and readiness to implement LAI-ART and learned that enthusiasm about the innovation was moderated by perceptions about the additional resources required to support patients in effectively switching to an injectable treatment formulation.
- This study identifies salient implementation determinants as well as implementation mechanisms and provides insight into how to overcome challenges.

## Background

The first long-acting injectable antiretroviral therapy(LAI-ART) to treat HIV was approved by the US Food and Drug Administration (FDA) in January 2021 [1–5]. This two-drug regimen consists of cabotegravir and rilpivirine (Cabenuva, ViiV Healthcare) administered as two gluteal injections every 4–8 weeks in a clinical setting. LAI-ART is indicated for use in individuals who are virologically suppressed on a stable oral ART treatment, are not known or suspected to be drug resistant to cabotegravir or rilpivirine, and have no history of treatment failure [6]. LAI-ART broadens patient choice and may be beneficial for those seeking an alternative to a daily oral pill. Furthermore, it may improve adherence [7] as has been the case in long-acting formulations of birth control, and antipsychotic medications [8–10].

HIV treatment has evolved substantially over the last 35 years [11, 12]. Initially, adherence was complicated by harmful, pervasive side effects, high pill burden, and a complex dosing schedule associated with the earliest therapeutic regimens [12]. With new developments in treatment, rates of optimal antiretroviral adherence increased as medication tolerability improved, and dosing schedules were simplified [13, 14]. Yet even with more forgiving regimens such as one pill, once a day, studies show that an estimated 42–60% of people living with HIV have poor to suboptimal adherence [15]. The barriers to medication adherence have been well outlined and include physical attributes such as difficulty swallowing pills, pill aversion, nausea, and other side effects and psychosocial and structural barriers such as stigma, homophobia, transphobia, racism, medical mistrust, housing instability, economic marginalization, and substance use disorder [16–26].

Theoretically, a subset of these obstacles could be addressed in the context of long-acting injectable ART. The monthly or bi-monthly dosing schedule of LAI-ART alleviates the burden of a daily routine, reduces stigma associated with possessing medication bottles and the injectable route of administration alleviates difficulties with swallowing medication. Realistically, unforeseen barriers to LAI-ART are likely to surface. The implementation science literature on other long-acting medications such as long-acting reversible contraception (LARC) or antipsychotic treatment, suggests that LAI-ART implementation efforts may falter at the system and provider levels. For example, the primary care health system remains unprepared to offer LARC services in part because there are too few primary care providers trained in the technique of inserting and removing the devices. Lack of training opportunities, particularly hands-on training, contributes to deficits in provider education and competency [27, 28]. Technical issues, e.g., no access to autoclave and logistical barriers related to time constraints, and billing concerns exist as well [29, 30]. Similar challenges arise in the implementation of injectable antipsychotic medications. Studies show low provider buy-in, lack of clear guidelines on initiating long-acting medication, difficulty integrating long-acting antipsychotics into clinic flow, and deficiencies in patient education and counseling [31, 32].

With the advent of long-acting injectable HIV antiretroviral therapy, an essential research question is how best to implement it to optimize its effectiveness, and how to do so in HIV clinic contexts where resources are constrained [33, 34]. Logically, using implementation science to address the research-to-practice gap [35, 36] can facilitate the design of clinic delivery systems that enable effective use of LAI-ART. Thus, we sought to assess key clinic stakeholder (i.e., staff, providers) perspectives early, during the pre-implementation phase, because implementation climate and readiness for change are antecedents to downstream implementation outcomes [37, 38]. Ideally, this formative research will enable us to identify key clinic-level actions needed to prepare for the successful roll-out of LAI-ART, a complex innovation, and optimize its potential public health impact [21, 23, 39].

## Methods

We conducted a qualitative, descriptive study as part of MODERN ART, a multi-site, mixed methods project evaluating the use of LAI-ART in urban HIV clinics caring for underserved people living with HIV (PLWH) in three US cities: San Francisco, Chicago, and Atlanta (Table 1). Our team leveraged the Consolidated Framework for Implementation Research [37] (CFIR) to study pre-implementation conditions in each clinical setting, e.g., resources, readiness, and feasibility. The CFIR is a determinant implementation science framework; it is widely used to illuminate modifiable structural or individual factors (determinants) that can facilitate or undermine successful intervention implementation [40–42]. CFIR consists of five multi-level, overarching domains: intervention characteristics, outer setting, inner setting, characteristics of individuals, and process. These domains are then associated with 39 discrete constructs and subconstructs such as relative advantage, complexity, and readiness for implementation. The “Process” domain was not explored given the pre-implementation phase of the study.

**Table 1:** Clinic characteristics

Site	Payer source	Medicaid expansion state	# PLWH served	Patient population	% patients virally suppressed
Site 1	Public insurance or uninsured (safety-net HIV clinic)	Yes	2500	85% cisgender men 13% cisgender women 2% transgender [wo]men	~70%
Site 2	Private or public insurance	Yes	600	63% cisgender men 37% are cisgender women <1% transgender [wo/men]	~80%
Site 3	Public insurance or uninsured (safety-net HIV clinic)	No	>6000	75% cisgender men 25% cisgender women <1% transgender [wo/men]	~70%

We conducted focus group discussions (FGDs) among key stakeholders in the three sites. We intentionally designed the groups to consist homogenously of either clinic staff or providers to minimize workplace power dynamics and encourage openness in sharing one’s opinions. Clinic staff members included nurses, pharmacists, clinic managers, social workers, and patient navigators. Provider groups included medical doctors, nurse practitioners, and physician’s assistants.

In consultation with a physician-researcher lead in each clinic, the team developed a list of providers and staff who would likely be involved in the delivery of LAI-ART programs. The research team emailed these potential participants and invited them to participate in a focus group discussion. Participants provided informed consent prior to data collection and were offered an honorarium of $50. The University of California, San Francisco Institutional Review Board, approved all study activities.

All FGDs were conducted remotely, audio-recorded, and moderated by KK, the lead author and Investigator. A research coordinator local to the site served as a note-taker. The research coordinator tracked the content of the conversation by speaker and monitored setting events, e.g., a participant going on or off camera. Each group lasted approximately 60 min and began with a brief description of LAI-ART and a set of guidelines to facilitate the discussion, e.g., “freely express your opinions” and “we encourage multiple points of view,” [43] followed by questions from a semi-structured moderator’s guide.

The guide was informed by the CFIR and developed iteratively in collaboration with KK and KC with input from other MODERN ART research team members. After the first FGD, we modified and narrowed the line of inquiry to the following major questions: “What do you think about LAI-ART for HIV?” (intervention and individual characteristics) “What are your concerns about LAI-ART?” (intervention characteristics, outer and inner setting) “What do you think it’s going to take to successfully launch LAI-ART?” (inner setting, individual characteristics) “What practical structures would ideally be put in place prior to offering LAI-ART?” (inner setting) “What existing aspects of the clinic would facilitate the delivery of LAI-ART?” (inner setting). We did not develop specific questions related to the 30 constructs nested within the 4 domains covered in our moderator’s guide. Instead, we anticipated that within each CFIR domain, the most salient constructs would spontaneously emerge.

Prior to arriving to the focus group, each participant self-completed a short, structured questionnaire about implementation readiness for implementing change. The questionnaire consisted of 15 questions, e.g., “Once the FDA approves long-acting injectable ART, the people who work here will do whatever it takes to figure out how implement.” Or “Once the FDA approves long-acting injectable ART, the people who work here will feel confident that they can handle the challenges might arise in implementing LAI-ART.” Other topics covered in the questionnaire included implementation intentions, organizational readiness for change, implementation of innovations, and personal demographics [44]. The moderator drafted a debriefing note following each FGD for distribution to the larger research team for discussion during weekly meetings.

A subset of the team (XE, KK, KC) developed the approach to data analysis (informed by the Krueger’s long table approach [43]). XE read across the focus group notes, verbatim transcripts, and debriefing notes to develop three site-specific memos that highlighted the themes that evolved at each site. KK and KC reviewed these site-specific memos; KK added additional details and expanded interpretations of the salient ideas identified by XE. All transcripts were imported into the qualitative data analysis web-based platform Dedoose [45]. XE applied a set of broad deductive and inductive codes to facilitate the retrieval of key quotes. The deductive codes generated by XE and KK were based on domains taken from the moderator’s guide, e.g., implementation concerns and implementation considerations. During the initial process of coding, XE identified narratives that were suited to inductive codes, e.g., “equity,” “getting comfortable with prescribing LAI-ART,” and “role of pharmacy.” Working with the memos as well as Dedoose code reports on equity, implementation considerations, implementation concerns, patient support, and retention, KK summarized coded excerpts and identified exemplar quotes. KK mapped data interpretations to CFIR constructs and shared these with the team for review and discussion. Following receipt of the team feedback, KK refined the interpretations and themes present in the data.

This study meets the standards for methodological rigor and excellence in qualitative research as laid out by Tracy [46]. Our topic is considered worthy, and the findings are credible and meaningfully coherent. The study team consists of practicing physicians who can attest to the importance and transferability of our findings to their peers. Numerous members of our study team have a history of conducting qualitative research that has made a significant contribution to the field of engagement in HIV care [26, 47–49].

## Findings

Between September 2020 and April 2021, we conducted 10 FGDs (5 with medical providers and 5 with clinic staff) with a total of 49 participants (Table 2). Characteristics of the study sample are presented in Table 3. Each FGD included between 3 and 7 individuals.

**Table 2:** Sample size and composition of focus group discussions by site

Clinic	# of FGD	Staff sample/type	Provider sample/type	Total
Site 1	3	*n*=9 Registered nurses Pharmacist Social worker	*n*=4 Medical doctors Nurse practitioner	*n*=13
Site 2	3	*n*=4 Registered nurses Pharmacist Social worker	*n*=11 Medical doctors Nurse practitioner	*n*=15
Site 3	4	*n*=7 Registered nurses Pharmacist Social worker Navigator Case manager	*n*=14 Medical doctors Nurse practitioners Physician assistant	*n*=21
Total	10	*n*=20	*n*=29	*n*=49

**Table 3:** Characteristics of medical providers and staff

	Sample *N* = 49
	*N* (%)
Gender	
Cisgender man	18 (36.7)
Cisgender woman	31 (63.3)
Race/ethnicity	
Asian	6 (12.7)
Pacific Islander/Hawaiian	1 (2.1)
Black/African American	10 (21.3)
White	27 (57.4)
Multiracial	2 (4.3)
Unknown	1 (2.1)
Latine	1 (2.1)
How far out from school/training are you?	
<5 years	13 (26.5)
5–15 years	14 (28.5)
>15 years	19 (38.7)
Currently in school/training	3 (6.1)
How long have you been caring for PLWH?	
Refused	1 (2.0)
<5 years	14 (28.5)
5–15 years	16 (32.6)
>15 years	18 (36.7)
Do you prescribe ART?	
No	20 (40.8)
Yes	29 (59.2)

**Fig. 1:**
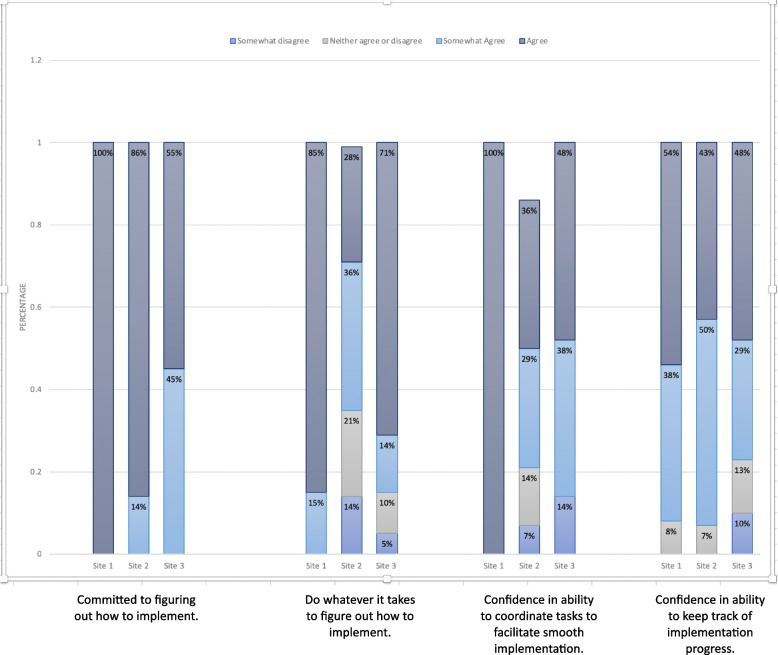
Organizational readiness for implementing change

**Fig. 2:**
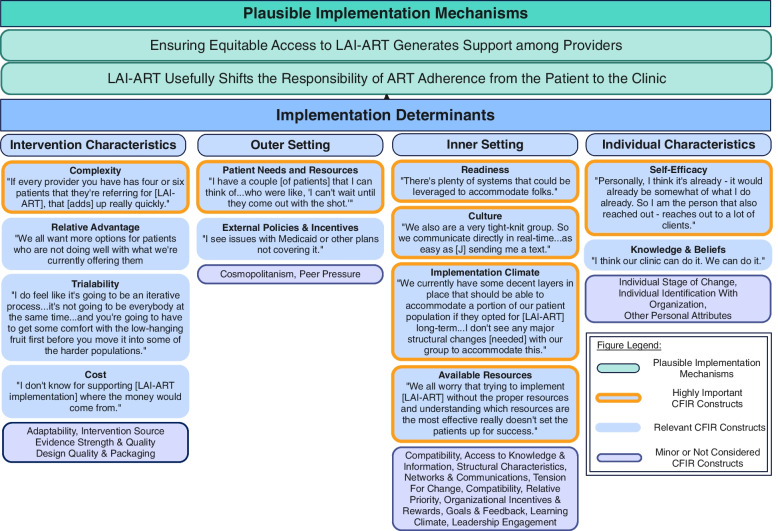
Findings mapped to CFIR domains and constructs

Quantitative findings from the questionnaire on implementation attitudes are presented first (Fig. 1). While participant responses were influenced by the unique inner and outer resources available in each clinic setting, we noted similarities related to the most impactful CFIR domains and constructs and modest differences between the provider and staff cohorts (Fig. 2). Next, we present qualitative findings regarding two major themes on implementation mechanisms. We then describe implementation determinants, including proposed strategies to address those that might be problematic.

### Results from the questionnaire on organizational readiness

Questionnaire results from the organizational readiness for implementing change were closely aligned with the high levels of acceptability of LAI-ART stated during the FGDs. All of the medical providers (*n*=29) indicated that they planned to prescribe LAI to at least one current patient. Among staff and providers, 84–100% “somewhat agreed” or “agreed” with being committed to figuring out how to implement LAI-ART and ‘doing whatever it takes’ to offer LAI. A similarly high proportion of 86–88% staff and providers reported feeling confident that they could coordinate tasks for smooth implementation and keep track of implementation processes Fig. 1.

### Findings from the focus group discussions

#### Implementation mechanism: ensuring equitable access to LAI-ART will generate support among providers

Overall, attitudes toward LAI-ART were positive. There was consensus on the perceived benefits of an alternative to daily oral ART and a shared sentiment that offering patients a choice in medication modalities was advantageous, e.g., “options are always better for our patient population.” Participant belief in the efficacy of the LAI-ART intervention contributed to the excitement about its value, and although concerns about the possibility of drug resistance were raised by a few providers, they did not significantly dampen this enthusiasm. At the time of these FGDs, the FDA approval process was underway, and the indicated use of LAI-ART was for individuals who were virally suppressed, had no known drug resistance, and no treatment failure. As participants reckoned with the fact that patients less-well engaged in care, such as those who were viremic or had a history of treatment failure, might not be eligible for LAI-ART as labeled, their enthusiasm for and acceptability of LAI-ART diminished.

In nearly every focus group, the first individual to respond to the initial question (What do you think about long-acting injectable ART?) offered an affirming attitude — “I think it’s awesome.” By the time the third person offered their opinion, however, the discussion had typically shifted away from the positive aspects of LAI-ART towards areas of concern; conversations went from “it’s great” to “it’s great, but….”. This shift was almost always motivated by questions about the equitable distribution of LAI-ART. Participants raised concerns about the potential for unequal impact across patient populations –– “which patients will benefit from this intervention the most? Will the selection process be equitable?” Enthusiasm for LAI-ART thus appeared to be contingent on whether participants perceived that there was an equitable path forward for this novel HIV treatment formulation. The following excerpt taken from a focus group with providers illustrates how they built off one another’s point of view while discussing the drawbacks of LAI-ART:

P4: …like everyone else, I’m really excited about Cabenuva for our patients. . . but there’s a really big subset of patients who don’t meet those criteria that would potentially benefit from long-acting therapy.

P5: I completely agree with that point. I feel like it’s another example of sometimes penalizing patients because of their HIV. … I do think that my patients who are hearing they’re not eligible because you can’t take a pill is again another stigmatizing thing we’re doing to HIV patients. – Site 3 Provider

Participants went on to discuss the types of patients who had expressed interest in LAI-ART, noting that many were those who struggled with adherence rather than those who were perfectly adherent. The conversation returned to an overwhelming recognition of the inconsistency between the type of patient the new drug delivery platform would benefit and the type of patient for whom the medication was indicated:

. . .the whole purpose of this medication delivery system was to decrease a barrier, and then at the same time we’re being told, “Oh, we’re going to use this patient’s barriers that they already have against them for a medication that’s designed to deal with those barriers. It’s a little, like she said, like an oxymoron in a way. It’s a little paradoxical. – Site 3 Provider

One participant noted a similar trajectory in the case of their clinic’s implementation of rapid ART initiation whereby individuals newly diagnosed with HIV are offered HIV treatment on the day of diagnosis. Implementation of rapid ART lagged when providers were reluctant to give up (or de-implement) the usual practice to look for signs of patient readiness to initiate ART rather than assuming every patient may be ready to start HIV treatment upon being diagnosed. With this history in mind, the participant suggested that the conservative 'mindset' about the target audience for LAI-ART would change over time, as it had (in their experience) with rapid ART. This mindset was said to be an organic part of a “learning curve” related to figuring out how to implement LAI-ART and a part of the typical “growing pains” associated with implementing an innovation Fig. 2.

#### Implementation mechanism: LAI-ART will usefully shift the responsibility of ART adherence from patients to the clinic

Providers and staff articulated a subtle, but potentially consequential shift brought on by the introduction of LAI-ART. With LAI-ART, the responsibilities associated with adherence are largely redistributed from the patient to the clinic. With oral formulations, providers and staff play a critical, but somewhat peripheral role in supporting adherence. They are the conduit to ART prescriptions and may engage in adherence counseling and/or provide support, e.g., pillboxes and check-ins, as needed. However, the procurement, monitoring of medication supply, and dosing largely default to being the responsibilities of the patient. In contrast, LAI-ART generally requires clinics to procure, store, administer, and track the administration of the medication.

In some groups, participants discussed the unique obligations LAI-ART would entail. They recognized the possibility for and wanted to guard against “dropping the ball” when it came time to implement LAI-ART. Specifically, they wanted to avoid scheduling and medication-procurement or storage errors. When asked about what existing structures might facilitate implementation, we learned that any existing process or system would require strengthening.

The existing structures are somewhat looser than they probably should be. . .we wouldn’t be doing our patients any service if we were the ones dropping the ball if we know that patients are going to need comprehensive follow-up to make this work for them. – Site 1 Staff

Because LAI-ART must currently be administered by a medical professional, new workflows and processes must be developed for clinics to offer LAI-ART. Some staff, especially nurses, described this transfer of responsibility as a new opportunity to be more involved in adherence support. Others framed it as more of a burden — LAI-ART *requires* providers and staff to become centrally involved in adherence, e.g., through scheduling, tracking, and administering injections and careful follow-up for missed injections. Yet regardless of whether LAI-ART implementation was perceived as an opportunity or a burden, it was no longer just the patient who needed to be organized around adherence behavior and support, but also the clinic. Otherwise, as one participant stated, “…if we’re not all organized about this, it will go wrong.”

Many providers and staff acknowledged that changes at the clinic level would be essential to make the most of this innovation. For example, strategies and workflows to proactively support patients to attend monthly or bi-monthly injection visits would be needed. Under the current system, most clinics are designed to “wait for patients to come…” Regarding LAI-ART, a passive approach could lead to adherence failure, particularly for patients in need of reminders or outreach to ensure timely attendance to injection visits. One participant expressed concern about the “onus” that LAI-ART may place on the workforce to “stay on top of patients more,” a task that is particularly difficult in safety-net clinics serving disenfranchised patients.

The concern I have is the patients that are going to benefit from this the most are going to be the hardest ones to reach and keep coming back in every two months. And so, I think in order to obtain that equity that [Provider X] referred to is going to require a lot of surveillance and effort on the part of the provider to really try to keep patients on task. And that’s the one thing that worries me, is I feel like this might put more onus and more workload on the physician and health care workforce to really try to stay on top of these patients more. That being said, I think if that’s done well, it could be very beneficial for our patients. Especially the ones that are challenged to be adherent. – Site 2 Provider

Perceptions of implementation determinants otherwise known as implementation barriers and facilitators depended on the degree to which (1) existing clinic structures could be built upon and (2) the adaptative capacity of the clinic and individuals within it could be leveraged. These issues are discussed below.

#### Implementation determinants

##### Intervention characteristics

The CFIR domain “intervention characteristics” in this study refers to the innovation of LAI-ART treatment for HIV.

*Relative advantage*: Participants often initiated the discussion by pointing to the advantages of LAI-ART as an exciting alternative to daily oral treatment. These advantages are often related to a perception that it would enhance the quality of life for patients experiencing pill fatigue and/or HIV-related stigma since some patients keep their medication hidden. LAI-ART would also be advantageous for patients who had difficulty taking a daily pill but had no difficulty attending scheduled HIV care visits. However, participants expressed that there was no relative advantage for patients who currently attend 2–3 clinic visits per year, as LAI-ART would require as many as 6–12 visits per year. Nor would it be advantageous for patients who were satisfied with their existing regimen or had a prohibitive fear of needles. Further discussion of the merits of LAI-ART led to conclusions that the “straightforward” advantage would be a quality-of-life improvement, but only for some patients:

There’s the population who’s pretty virologically suppressed. And this’ll be a really nice break from daily pills, but it doesn’t really change the universe as far as our ability to control the epidemic, but it may change those individuals’ lives. – Site 3 Provider

Provider acknowledged that given the current FDA indications limiting on label use to those with no history of virologic failure, LAI-ART would not lead to the end of the HIV epidemic. One provider predicted that such a paradigm shift would occur only once systems and structural issues were developed to serve viremic patients. Until then, there was skepticism that LAI-ART could significantly impact the HIV epidemic.

I mean from a public health and global perspective, [LAI-ART] will enhance the quality of life for a subset of individuals, and that’s great. The big paradigm shift will be when these drugs become approved for the untreated population. – Site 1 Provider

*Complexity*: This construct is defined as the extent to which LAI-ART was perceived to be difficult to implement. Discussions about complexity were almost always intertwined with references to existing resources or lack thereof (an Inner Setting construct). Importantly, participants did not consider LAI-ART implementation to be highly complex for well-engaged patients. The perception was that supporting this type of patient would not necessarily create added strain on the clinical system because they had already proved their reliability by being adherent to routine medical appointments and being virally suppressed.

I think there are a group of people that would look forward to [LAI-ART]. And who would participate without too much extra effort on our part. I think where we’ve ran into trouble are dealing with the patients that … don’t necessarily come in now. ….And I think that’s where most of the work will be. – Site 3 Provider

*Trialability*: Participants reported the notion of LAI-ART as an innovation that could and should be trialed or tested on a small scale. A common theme across groups and cohorts was that a “trial run” of LAI-ART implementation was essential and that patients adherent to oral ART and appointments were the best patients with whom to test new procedures. Whether clinics could move beyond the trial period was a key question in 2 of the 3 sites. One participant calculated that implementation complexity would be manageable up to a point — basically, manageable until it was not. If each provider in a clinic with 10 providers recommended LAI-ART to one or two patients the total number of patients requiring tracking and monitoring would be 10–20. Beyond that, they stated: “It’s going to get complicated quick. And we don’t have a lot of personnel to lean on.” This point was echoed by a provider in Site 3 who stated: “…if we have ten patients, it’s one thing. But if we have even 5%, I think it’s, that’s 300 patients. So, scale is always our challenge.”

##### Outer setting

The CFIR domain “outer setting” refers to “the economic, political, and social contexts of each of the participating clinics [37].

*Patient needs and resources*: In every focus group, the driving force underlying the acceptability of LAI-ART was its potential value to improve the lives of patients. The providers and staff expressed a very high level of awareness about patient needs and especially emphasized the needs of the most underserved populations, e.g., those experiencing substance use disorders and/or homelessness. Participants easily enumerated the barriers that could be resolved with a long-acting treatment formulation, ranging from managing internalized HIV stigma “some days they just don’t take it because they don’t want to think about their HIV” to those coping with structural violence:

We deal with a big homeless population, and a lot of the stories I hear is that they lose their medication or they’re stolen. So, I think it’d be great for them…– Site 3 Staff

Providers reported talking with some of their patients about long-acting formulations, but until implementation ensued, questions critical to effective implementation remained: Who is going to pay for the medications? Will it be a pharmacy or medical benefit? How many patients want LAI-ART?

##### Inner setting

The CFIR domain “inner setting” refers to the “structural, political, and cultural contexts where the implementation will take place” [37].

*Readiness, Implementation Climate, Available Resources*: It was difficult to identify the boundary between narratives that emphasized a sense of individual self-efficacy to participate in implementation of LAI-ART and narratives evoking collective-efficacy among the “we.” In nearly all staff focus groups, the positive regard for long-acting injectable medications manifested in a ‘we will find a way’ sensibility. One staff person stated, “Once tools [are] there, we’ll make the means to make it happen.” Overall, staff consensus was that they would be able to offer LAI-ART to patients, and to do so, they simply needed to develop a system to make it work. The “we’ve got to do it” sentiment applied even if that meant more work on systems and staff to accomplish this goal.

We detected similarities in general enthusiasm about LAI-ART across staff and provider cohorts, but notable differences in opinion about the degree of burden LAI-ART implementation would pose, namely on staff. Across sites, providers routinely expressed concerns about the ability and willingness for nurses, pharmacists, and social workers to manage the work associated with rolling out LAI-ART. Providers were similarly pessimistic about the capacity for nursing staff to take on added responsibilities associated with a new LAI-ART program, e.g., “our nurses may not have the bandwidth” or “[it’s] more than our staff can absorb right now.” Meanwhile, staff were aware of the extra work LAI-ART implementation entailed but were generally eager to participate.

In all sites, the view was that while providers would be integral to uptake, i.e., discussing the option with patients, implementation of LAI-ART would likely be executed by staff and pharmacists, not by providers. Thus, it was notable that staff narratives were routinely optimistic as illustrated by a nurse manager below:

…something like that is feasible in our clinic setting. We can develop a workflow to accommodate that patient population that needs monthly injections. . . And then, if we get a significant amount of patients that qualify and want [LAI-ART], then, we can redesign our workflow to accommodate that. – Site 2 Staff

In sites 1 and 2, we noted a sense of optimism and faith in the existing clinic *culture* to make implementation of LAI-ART work such as “our clinic is historically very good at overcoming difficult challenges.” Confidence in clinic culture/readiness to implement was not as present in site 3 — despite expressing high levels of acceptability, the perception of feasibility of implementing LAI-ART was low. The level of pessimism or optimism related to implementation feasibility was influenced by the availability of resources. Participants in site 2 expressed the greatest level of confidence in their availability of resources. And participants in site 3 were the most fatalistic about their chances of scaling up LAI-ART beyond a small pilot.

We are understaffed and overworked and don't have nurses and don't have a layout like other clinics who are well funded and they have resources to provide the staff that they need to run clinics smoothly.– Site 3 Provider

#### Individual characteristics

The CFIR domain “individual characteristics” refers to the “roles and characteristics of the individuals” [37] working within the implementation space. We identified “self-efficacy” and “knowledge and beliefs” as relevant constructs; however, because they were so closely related to the “inner setting” constructs of “culture” and “implementation readiness,” we discussed these above.

#### Recommendations for moving implementation forward

Our research suggests that all sites could support the implementation of LAI-ART with a limited number of patients using existing resources and that additional resources would be necessary to scale up implementation, particularly to provide adherence support to patients coping with significant obstacles to consistent engagement in HIV care. Conversations about implementation complexity typically led to discussions about how the clinic might prepare to navigate potential challenges. Participants outlined ideas about how to optimize LAI-ART implementation. Our distillation of the proposed strategies include methods to address inner setting deficiencies to foster readiness (1–8) and solutions to promote equitable distribution of LAI-ART (9–11): (1) promote opportunities to hear from peers with experience implementing LAI-ART; (2) provide LAI-ART education to providers, staff and patients; (3) assemble a dedicated, centralized LAI-ART team and assign clear roles along with a manageable workload, e.g., provider-champion, pharmacist, nurse, social worker; (4) adopt useful models or tools from other injection clinics; (5) launch a pilot with a few patients; (6) develop concrete patient eligibility criteria; (7) develop a LAI-ART patient tracking system to display information about scheduled and missed injection visits; (8) create an outreach plan with assigned roles for clinic staff in the event of a missed injection appointment; (9) offer scheduled rather than drop-in injection visits, but accommodate drop-ins when possible; (10) consider utilizing incentives; (11) consider a workflow to bring LAI-ART to the patient or develop an after-hours injection clinic.

## Discussion

Among providers and staff in three urban clinics caring for underserved PLWH, we found high levels of acceptability of LAI-ART, as evidenced by a sense of enthusiasm about its potential to improve quality of life and to potentially resolve barriers to daily oral ART adherence. We also documented concerns about the equitable distribution of LAI-ART, concerns about the potential strain of implementation on existing resources, and questions about logistics and payer source.

Our study echoes other research reporting that providers in similar settings in the US “generally support” or find LAI-ART to be acceptable [50, 51]. We also report on concerns that have been documented in the literature though we do so using an implementation science framework. Shared concerns about LAI-ART implementation fell primarily under the outer and inner setting domains including payer source [52] or insurance concerns [51], staff and clinic system capacity to support injections [53], monitoring appointments [53, 54], and managing missed appointments [54]. In their review article on phase 3 clinical trial results and implementation considerations, authors Bares and Scarsi [53] reported provider concern about patient adherence to injection visits, but it was not clear if the concern was an expression of fatalism about the inevitability of patients to miss visits or if the concern arose from deficiencies in clinic systems to adequately support patients to routinely attend injection visits or a combination of the two. Our data supports a combination of anxiety about patients missing injection appointments and the perceived effort clinics would need to shoulder to adequately support patient adherence to injection visits. Notably, patient adherence to visits was not necessarily a chief barrier expressed in the literature on long-acting injectable contraception or antipsychotic medication.

One of the implicit goals of our pre-implementation research was to identify how the perspectives of LAI-ART among providers and staff would contribute to or work against the successful downstream implementation of this new treatment option. Assessing implementation through the lens of the CFIR provided us with insights related most saliently to the intervention characteristics construct of *complexity*. The complexity of the LAI-ART intervention echoed throughout the focus group discussions as participants volleyed two distinct polarities, from little complexity, expressed as “not too much effort on our part” to a lot of complexity, expressed as “it’s going to get complicated quick.” For less-well engaged patients the complexity of implementing LAI-ART was expected to lead to more intensive work by the clinic. The expected outcomes of which could yield major benefits for patients who had been unable to consistently adhere to daily oral ART.

The amount of work necessary to effectively outreach to help less-well engaged patients adhere to a timely injection schedule every month was hard to quantify. Participants could only surmise what that output might look like based on past experiences. To be sure, this is an important question to ask and answer in future programs. Until proven otherwise, the tacit hypothesis seemed to be that engaging patients who face numerous barriers to adherence would make the implementation of LAI-ART challenging and more complex than for those patients with no history of adherence issues. It will be important to evaluate the assumption that the implementation of LAI-ART for highly adherent patients will be uncomplicated. Emerging data from at least one study reporting on implementation outcomes among highly adherent patients suggests otherwise [55]. This study reported “substantial human capital” was necessary to attain and administer the medication and to support patients using LAI-ART. The degree to which patient-staff ratios impact efforts to implement LAI-ART will be important to attend to.

Our use of the CFIR allowed us to “catalogue” key constructs at play in the LAI-ART implementation environment [56]. However, determinant frameworks, such as CFIR, are deployed in a static fashion. Their strengths lie in being able to enumerate the anatomy of an implementation context, but conversely, they are not well positioned to explain the physiology of said context [56, 57]. Thus, our use of CFIR did not facilitate our understanding of how key constructs *interacted* with one another. Because we wanted to push beyond a static perspective and offer an explanation about the relationships between determinants, we looked beyond the CFIR. Our analysis of mechanisms that might predict implementation outcomes allowed us to theorize that the successful rollout of LAI-ART requires ensuring equitable access to LAI-ART within clinic settings that are optimized to take on the myriad responsibilities associated with medication adherence. This theory may be tested in future studies.

We documented important implementation determinants that would likely facilitate LAI-ART implementation. Many participants expressed self-efficacy in their own abilities to effectively participate in LAI-ART implementation. These sentiments were clearly reflected in the results of the brief survey which showed high degrees of confidence in the ability to implement. Staff, in particular, conveyed optimism about the absorptive capacity of the clinic to perform adherence support in the form of ongoing surveillance and outreach associated with a LAI-ART service. Staff expressed a willingness to coordinate their work differently to accommodate the implementation requirements of LAI-ART, indicating that the return on investment would be well worth it. Literature suggests that when an evidence-based innovation has intrinsic appeal to prospective implementers, it is more likely to be implemented [58]. As proponents of LAI-ART, staff indicated they would strive to remove implementation barriers. If staff are successful in balancing their existing work with additional LAI-ART delivery tasks, theoretically the implementation climate will improve, and an improved delivery climate may impact other individuals in the setting. For example, those holding less favorable attitudes about the feasibility of implementing an innovation may relax their resistance. Research to assess staffing resources such as nurses and pharmacists during the implementation phase of LAI-ART could help determine the amount of effort needed to effectively scale the delivery of LAI-ART. For example, a study leveraging the Normalization Process Theory (NPT), a implementation science framework used to assess what it takes for an innovation such as LAI-ART to become embedded or integrated in practice, would be worthwhile [59, 60]. NPT is a dynamic theory and the use of this theory would provide critical information about the generative mechanisms that foster change and lead to implementation outcomes.

Future studies may also include a focus on understanding institutional absorptive capacity [61]. Given the patient populations served by the participating clinics, providers and staff were highly motivated to find ways to narrow the possibility of an equity gap between those with and without access to LAI-ART. Despite facilitating individual characteristics such as high self-efficacy to implement LAI-ART and a strong belief in the innovation, motivations could easily be dampened by a lack of material and human resources to absorb the very real work necessary to carry out an effective LAI-ART program. Future studies focused on LAI-ART implementation might incorporate the concept of absorptive capacity, the ability of an organization to effectively integrate and apply new knowledge, information, and innovations [62]. Our study suggests absorptive capacity is a fruitful approach to studying the success or failure of implementation efforts and as such is a logical starting point for future inquiries to better understand and account for absorptive capacity including its constituent parts and how these parts interact to produce effective uptake of LAI-ART [63].

## Limitations

This study is not without limitations. The differences in HIV healthcare financing across the three sites make each setting somewhat unique. Two of the three clinics serve publicly and privately insured patients; paradoxically, commercial insurance authorization processes may be more complicated than those of public insurance. All three settings share a designation as academic medical centers. We encourage future research in settings such as community clinics and federally qualified health centers or Ryan White-funded clinics that are unaffiliated with academic centers. Limitations also apply to our data collection methods. We initially planned to conduct focus groups in person and switched to a videoconferencing format due to public health restrictions during the height of the COVID-19 pandemic. The videoconference format prevented the moderator from using the time just prior to starting the group to informally build rapport with participants. In addition, the use of videoconferencing thwarted efforts for the moderator to gain firsthand exposure to the clinical setting (or inner context) where implementation of LAI-ART would take place. Relatedly, our sample has limitations. We recruited a subset of the providers and staff in each site and had we convened groups with all providers and staff in each site, we may have heard different opinions about the acceptability and feasibility of implementing LAI-ART. The possibility that participants provided socially desirable responses, in this case, to preserve professional reputations or job security, is a perennial issue for social and behavioral researchers. Yet this potential bias did not preclude candid responses about challenges to implementing LAI-ART. The focus groups were moderated by an individual without a clinical background and may not have sufficiently probed about specific medical concerns held by prescribers related to, for example, severe non-IgE mediated and IgE-mediated adverse drug reactions. Lastly, data were collected prior to FDA approval in two of the three sites. However, implementation of LAI-ART had not begun in Site 3 during our data collection period. All sites were in the pre-implementation phase.

The strengths of our study include the substantial involvement of clinician-researchers from each of the clinics, which meant that the research questions and analysis were narrowly focused on questions that are important and salient to individuals working in “real world” conditions. In addition, as a multi-site study, we had the advantage of studying contextual influences to assess how these may differ from one clinic to another.

Our findings may make a substantial contribution to future implementers who may wish to integrate future novel devices to deliver HIV ART such as an implant. Understanding that in the case of HIV clinics, the clinic setting and the individuals within the clinic have a profound influence on implementation efforts. Thus, assessing the readiness of the environment and the workforce within it can direct capacity-building efforts to prepare for forthcoming innovations and may speed up future implementation efforts. It remains to be seen whether these findings may be consequential for researchers working in fields outside of HIV disease.

## Conclusion

In this exploration of attitudes about the implementation of LAI-ART among providers and staff working in three geographically distinct settings, we found that providers want to offer LAI-ART to a variety of patients. Staff want to implement strategies to support providers to offer LAI-ART, encourage patients to adopt the new treatment modality, and persist with injections. The main challenges to implementation include concerns about unequal access and a recognition that strengthening the clinic system is critical. To bring the current formulation (provider-delivered injections every 4 or 8 weeks) of LAI-ART implementation to scale will require approaches that are initially, and perhaps consistently, resource intensive.

## Data Availability

Data sharing is not applicable to this article as no datasets were generated or analyzed during the current study.

## Abbreviations

ART: Antiretroviral therapy
CFIR: Consolidated Framework for Implementation Research
FDA: Food and Drug Administration
FGDs: Focus group discussions
FQHC: Federally qualified health center
HIV: Human immunodeficiency virus
LAI-ART: Long-acting injectable antiretroviral therapy
PLWH: People living with HIV

## References

[1] Orkin C, Oka S, Philibert P (2021). Long-acting cabotegravir plus rilpivirine for treatment in adults with HIV-1 infection: 96-week results of the randomised, open-label, phase 3 FLAIR study. Lancet HIV.

[2] Orkin C, Bernal Morell E, Tan DHS (2021). Initiation of long-acting cabotegravir plus rilpivirine as direct-to-injection or with an oral lead-in in adults with HIV-1 infection: week 124 results of the open-label phase 3 FLAIR study. Lancet HIV.

[3] Jaeger H, Overton ET, Richmond G (2021). Long-acting cabotegravir and rilpivirine dosed every 2 months in adults with HIV-1 infection (ATLAS-2M), 96-week results: a randomised, multicentre, open-label, phase 3b, non-inferiority study. Lancet HIV.

[4] Margolis DA, Gonzalez-Garcia J, Stellbrink HJ (2017). Long-acting intramuscular cabotegravir and rilpivirine in adults with HIV-1 infection (LATTE-2): 96-week results of a randomised, open-label, phase 2b, non-inferiority trial. Lancet.

[5] Overton ET, Richmond G, Rizzardini G (2020). Long-acting cabotegravir and rilpivirine dosed every 2 months in adults with HIV-1 infection (ATLAS-2M), 48-week results: a randomised, multicentre, open-label, phase 3b, non-inferiority study. Lancet.

[6] Viiv Healthcare (2021). Cabenuva® prescribing information.

[7] Spreen WR, Margolis DA, Pottage JC (2013). Long-acting injectable antiretrovirals for HIV treatment and prevention. Curr Opin HIV AIDS.

[8] Winner B, Peipert JF, Zhao Q (2012). Effectiveness of Long-Acting Reversible Contraception. N Engl J Med.

[9] Curtis KM, Peipert JF (2017). Long-Acting Reversible Contraception. N Engl J Med.

[10] Titus-Lay EN, Ansara ED, Isaacs AN, Ott CA (2018). Evaluation of adherence and persistence with oral versus long-acting injectable antipsychotics in patients with early psychosis. Mental Health Clin.

[11] Forsythe SS, McGreevey W, Whiteside A (2019). Twenty years of antiretroviral therapy for people living with HIV: global costs, health achievements economic benefits. Health Aff.

[12] Tseng A, Seet J, Phillips EJ (2015). The evolution of three decades of antiretroviral therapy: challenges, triumphs and the promise of the future. Br J Clin Pharmacol.

[13] Franco M, Diego R, Claudio A (2002). Simpler regimens may enhance adherence to antiretrovirals in HIV-infected patients. HIV Clin Trials..

[14] Youn B, Shireman TI, Lee Y, Galárraga O, Wilson IB. Trends in medication adherence in <scp>HIV</scp> patients in the <scp>US</scp> , 2001 to 2012: an observational cohort study. J Int AIDS Soc. 2019;22(8). 10.1002/jia2.25382. 10.1002/jia2.25382 PMC6706701 31441221

[15] McComsey GA, Lingohr-Smith M, Rogers R, Lin J, Donga P (2021). Real-world adherence to antiretroviral therapy among HIV-1 patients across the United States. Adv Ther..

[16] Golin CE, Liu H, Hays RD (2002). A prospective study of predictors of adherence to combination antiretroviral medication. J Gen Intern Med.

[17] Katz IT, Ryu AE, Onuegbu AG (2013). Impact of HIV-related stigma on treatment adherence: systematic review and meta-synthesis. J Int AIDS Soc..

[18] Rintamaki LS, Davis TC, Skripkauskas S, Bennett CL, Wolf MS (2006). Social stigma concerns and HIV medication adherence. AIDS Patient Care STDS.

[19] Rintamaki L, Kosenko K, Hogan T (2019). The role of stigma management in HIV treatment adherence. Int J Environ Res Public Health..

[20] Turan B, Rice WS, Crockett KB (2019). Longitudinal association between internalized HIV stigma and antiretroviral therapy adherence for women living with HIV. AIDS..

[21] Palepu A, Milloy MJ, Kerr T, Zhang R, Wood E (2011). Homelessness and adherence to antiretroviral therapy among a cohort of HIV-infected injection drug users. J Urban Health.

[22] Creasy SL, Henderson ER, Bukowski LA, Matthews DD, Stall RD, Hawk ME (2019). HIV Testing and ART adherence among unstably housed black men who have sex with men in the United States. AIDS Behav.

[23] Teti M, Bauerband LA, Altman C (2019). Adherence to antiretroviral therapy among transgender and gender nonconforming people living with HIV: findings from the 2015 U.S. Trans Survey. Transgend Health.

[24] Galvan FH, Bogart LM, Klein DJ, Wagner GJ, Chen YT (2017). Medical mistrust as a key mediator in the association between perceived discrimination and adherence to antiretroviral therapy among HIV-positive Latino men. J Behav Med.

[25] Pellowski JA, Price DM, Allen AM, Eaton LA, Kalichman SC (2017). The differences between medical trust and mistrust and their respective influences on medication beliefs and ART adherence among African-Americans living with HIV. Psychol Health.

[26] Wood TJ, Koester KA, Christopoulos KA, Sauceda JA, Neilands TB, Johnson MO. If someone cares about you, you are more apt to come around: Improving HIV care engagement by strengthening the patient–provider relationship. Patient Prefer Adherence. 2018;12. 10.2147/PPA.S157003. 10.2147/PPA.S157003 PMC5973398 29872277

[27] Phillips J, Sandhu P (2018). Barriers to implementation of long-acting reversible contraception: a systematic review. J Am Assoc Nurse Pract..

[28] Murphy MK, Stoffel C, Nolan M, Haider S (2016). Interdependent barriers to providing adolescents with long-acting reversible contraception: qualitative insights from providers. J Pediatr Adolesc Gynecol..

[29] Cowen L, Hartman SG, Loomis E, Srinivasan S, Gasbarro C, Young J. Clinician and Staff Perceptions of Barriers to Providing Contraception in Primary Care. PRiMER. 2019;3. 10.22454/PRiMER.2019.228141. 10.22454/PRiMER.2019.228141 PMC7205092 32537573

[30] Gilmore K, Hoopes AJ, Cady J, Amies Oelschlager AM, Prager S, Vander Stoep A (2015). Providing long-acting reversible contraception services in seattle school-based health centers: key themes for facilitating implementation. J Adolesc Health.

[31] Kane JM, McEvoy JP, Correll CU, Llorca PM (2021). Controversies surrounding the use of long-acting injectable antipsychotic medications for the treatment of patients with schizophrenia. CNS Drugs..

[32] Velligan DI, Sajatovic M, Sierra C (2021). A program to increase the appropriate use of long-acting injectable antipsychotic medications in community settings. Psychiatric Services..

[33] Mantsios A, Murray M, Karver TS (2021). Multi-level considerations for optimal implementation of long-acting injectable antiretroviral therapy to treat people living with HIV: perspectives of health care providers participating in phase 3 trials. BMC Health Serv Res.

[34] Howe ZW, Norman S, Lueken AF (2021). Therapeutic review of cabotegravir/rilpivirine long-acting antiretroviral injectable and implementation considerations at an HIV specialty clinic. Pharmacotherapy..

[35] Mallonee S, Fowler C, Istre GR (2006). Bridging the gap between research and practice: a continuing challenge. Inj Prev.

[36] Rohrbach LA, Grana R, Sussman S, Valente TW (2006). Type II Translation. Eval Health Prof.

[37] Damschroder LJ. CFIR Constructs. Implement Sci. Published online 2009

[38] Damschroder LJ, Aron DC, Keith RE, Kirsh SR, Alexander JA, Lowery JC (2009). Fostering implementation of health services research findings into practice: a consolidated framework for advancing implementation science. Implement Sci.

[39] Nalugwa T, Handley M, Shete P (2022). Readiness to implement on-site molecular testing for tuberculosis in community health centers in Uganda. Implement Sci Commun.

[40] Proctor EK, Powell BJ, McMillen JC (2013). Implementation strategies: recommendations for specifying and reporting. Implement Sci.

[41] Bauer MS, Damschroder L, Hagedorn H, Smith J, Kilbourne AM (2015). An introduction to implementation science for the non-specialist. BMC Psychol.

[42] Kirchner JAE, Smith JL, Powell BJ, Waltz TJ, Proctor EK (2020). Getting a clinical innovation into practice: An introduction to implementation strategies. Psychiatry Res.

[43] Krueger RA, Leader E (2006). Designing and Conducting Focus Group Interviews.

[44] Shea CM, Jacobs SR, Esserman DA, Bruce K, Weiner BJ (2014). Organizational readiness for implementing change: a psychometric assessment of a new measure. Implement Sci.

[45] Salmona M, Lieber E, Kaczynski D. Qualitative and Mixed Methods Data Analysis Using Dedoose: A Practical Approach for Research Across the Social Sciences. Sage Publications; 2019.

[46] Tracy SJ (2010). Qualitative Quality: Eight “Big-Tent” Criteria for Excellent Qualitative Research. Qual Inquiry.

[47] Koester KA, Johnson MO, Wood T, et al. The influence of the’good’ patient ideal on engagement in HIV care. PLoS One. 2019;14(3). 10.1371/journal.pone.0214636. 10.1371/journal.pone.0214636 PMC6438522 30921440

[48] Christopoulos KA, Olender S, Lopez AM, et al. Retained in HIV Care but not on antiretroviral treatment: a qualitative patient-provider dyadic study. PLoS Med. 2015;12(8). 10.1371/journal.pmed.1001863. 10.1371/journal.pmed.1001863 PMC4532493 26263532

[49] Christopoulos KA, Massey AD, Lopez AM (2013). “Taking a Half Day at a Time:” Patient Perspectives and the HIV engagement in care continuum. AIDS Patient Care STDS.

[50] Kerrigan D, Mantsios A, Gorgolas M, et al. Experiences with long acting injectable ART: A qualitative study among PLHIV participating in a Phase II study of cabotegravir + rilpivirine (LATTE-2) in the United States and Spain. PLoS One. 2018;13(1). 10.1371/JOURNAL.PONE.0190487. 10.1371/journal.pone.0190487 PMC5755771 29304154

[51] Kanazawa JT, Saberi P, Sauceda JA, Dubé K (2021). The LAIs are coming! Implementation science considerations for long-acting injectable antiretroviral therapy in the United States: a scoping review. AIDS Res Hum Retroviruses.

[52] Henegar C, South Lauren Collins UF, Collins LF, et al. Abstracts • OFID 2021:8 (Suppl 1) • S535 887. Implementation of Long-Acting Injectable Cabotegravir/Rilpivirine for HIV-1 Treatment at a Ryan White-Funded Clinic in The. https://academic.oup.com/ofid/article/8/Supplement_1/S535/6450514.

[53] Bares SH, Scarsi KK (2022). A new paradigm for antiretroviral delivery: Long-acting cabotegravir and rilpivirine for the treatment and prevention of HIV. Curr Opin HIV AIDS.

[54] Bogart L, Cohen R, Goodman-Meza D, et al. Preparing for Long-Acting Injectable Antiretroviral Therapy for HIV in Los Angeles Preliminary Findings Report UCLA Center for HIV Identification, Prevention and Treatment Services (CHIPTS) Study Team.

[55] Collins LF, Corbin-Johnson D, Asrat M, et al. Early Experience Implementing Long-Acting Injectable Cabotegravir/Rilpivirine for Human Immunodeficiency Virus-1 Treatment at a Ryan White-Funded Clinic in the US South. Open Forum Infect Dis. 2022;9(9). 10.1093/ofid/ofac455. 10.1093/ofid/ofac455 PMC9487705 36147599

[56] Kislov R, Pope C, Martin GP, Wilson PM (2019). Harnessing the power of theorising in implementation science. Implement Sci.

[57] Davidoff F (2019). Understanding contexts: how explanatory theories can help. Implement Sci.

[58] Brewster AL, Cherlin EJ, Ndumele CD (2016). What Works in Readmissions Reduction: How Hospitals Improve Performance. Med Care.

[59] May CR, Cummings A, Girling M (2018). Using Normalization Process Theory in feasibility studies and process evaluations of complex healthcare interventions: a systematic review. Implement Sci.

[60] Murray E, Treweek S, Pope C (2010). Normalisation process theory: a framework for developing, evaluating and implementing complex interventions. BMC Med.

[61] Christopoulos KA, Grochowski J, Mayorga-Munoz F, et al. First Demonstration Project of Long-Acting Injectable Antiretroviral Therapy for Persons With and Without Detectable Human Immunodeficiency Virus (HIV) Viremia in an Urban HIV Clinic. Clinl Infect Dis. 2022. 10.1093/cid/ciac631. 10.1093/cid/ciac631 PMC9907477 35913500

[62] Leung RC (2012). Health information technology and dynamic capabilities. Health Care Manage Rev.

[63] Innis J, Berta W (2016). Routines for change: how managers can use absorptive capacity to adopt and implement evidence-based practice. J Nurs Manag.

